# Macrolide resistance of *Mycoplasma pneumoniae* in several regions of China from 2013 to 2019

**DOI:** 10.1017/S0950268824000323

**Published:** 2024-04-18

**Authors:** Yue Jiang, Haiwei Dou, Bo Xu, Baoping Xu, Wei Zhou, Hong Wang, Lixia Ge, Yinghui Hu, Xiaohua Han, Xuanguang Qin, Jing Li, Leping Ye, Liqun Wu, Huimin Zuo, Qi Zhang, Ling Liu, Wenjuan Hu, Junyan Shao, Qiaomian Yin, Lina Han, Xiaoyan Fu, Xiaopei Dong, Yan Dong, Yulin Fu, Mengmeng Zhao, Qing Sun, Jingwei Huo, Die Liu, Wenkao Liu, Yunjuan Li, Yang Wang, Deli Xin, Kunling Shen

**Affiliations:** 1 Beijing Chaoyang Hospital,Capital Medical University, Beijing, China; 2 Tropical Medicine Research Institute, Beijing Friendship Hospital, Capital Medical University, Beijing, China; 3 Beijing Friendship Hospital, Capital Medical University, Beijing, China; 4 Beijing Children’s Hospital, Capital Medical University, China National Clinical Research Center of Respiratory Diseases, National Center for Children’s Health, Beijing, China; 5 Peking University Third Hospital, Beijing, China; 6 Civil Aviation General Hospital, Beijing, China; 7 China Meitan General Hospital, Beijing, China; 8 New Century International hospital for Children, Beijing, China; 9 Shengjing Hospital of China Medical University, Shenyang, China; 10 Beijing Changping District Integrated Traditional Chinese and Western Medicine Hospital, Beijing, China; 11 Peking University First Hospital, Beijing, China; 12 Dongfang Affiliated Hospital of Beijing University of Chinese Medicine, Beijing, China; 13 The First Hospital of Tsinghua University, Beijing, China; 14 China-Japan Friendship Hospital, Beijing, China; 15 Shenzhen Children′s Hospital, Shenzhen, Guangdong Province, China

**Keywords:** *Mycoplasma pneumoniae*, macrolide, drug-resistant, epidemic, clinical features

## Abstract

This paper retrospectively analysed the prevalence of macrolide-resistant *Mycoplasma pneumoniae* (MRMP) in some parts of China. Between January 2013 and December 2019, we collected 4,145 respiratory samples, including pharyngeal swabs and alveolar lavage fluid. The highest PCR-positive rate of M. pneumoniae was 74.5% in Beijing, the highest resistance rate was 100% in Shanghai, and Gansu was the lowest with 20%. The highest PCR-positive rate of *M. pneumoniae* was 74.5% in 2013, and the highest MRMP was 97.4% in 2019; the PCR-positive rate of *M. pneumoniae* for adults in Beijing was 17.9% and the MRMP was 10.48%. Among the children diagnosed with community-acquired pneumonia (CAP), the PCR-positive and macrolide-resistant rates of *M. pneumoniae* were both higher in the severe ones. A2063G in domain V of 23S rRNA was the major macrolide-resistant mutation, accounting for more than 90%. The MIC values of all MRMP to erythromycin and azithromycin were ≥ 64 μg/ml, and the MICs of tetracycline and levofloxacin were ≤ 0.5 μg/ml and ≤ 1 μg/ml, respectively. The macrolide resistance varied in different regions and years. Among inpatients, the macrolide-resistant rate was higher in severe pneumonia. A2063G was the common mutation, and we found no resistance to tetracycline and levofloxacin.

## Introduction

*Mycoplasma pneumoniae* (*M. pneumoniae*) is a common pathogen of respiratory tract infections in children, with an epidemic occurring every 3–7 years [1, 2], mostly in schools, military units, and other places with relatively high population concentrations, accounting for about 10–40% of children with community-acquired pneumonia (CAP) [3, 4]. Children and adults are susceptible to *M. pneumoniae*, and school-age and adolescent children have a higher incidence and are more likely to develop pneumonia or severe disease [5]. Macrolide-resistant *M. pneumoniae* (MRMP) was first isolated from clinical patients in Japan in 2000, after which the emergence of clinically resistant strains was reported in Asia, Europe, and North America, with different rates of resistance in different regions. The resistance rate of *M. pneumoniae* in China is at a high level, reaching more than 90% in specific regions, and is higher in children than in adults, which makes clinical treatment difficult. [6]

Our laboratory has conducted multicentre *M. pneumoniae* prevalence and macrolide-resistant surveillance since 2013. In the study, we conducted a retrospective study of *M. pneumoniae* clinical surveillance programmes in selected regions of China between 2013 and 2019 and statistically analysed macrolide resistance in age, gender, outpatient and ward, and severe/common pneumonia. What is more, we conducted a minimum inhibition concentration (MIC) of commonly used antibiotics. This paper aims to increase the knowledge of *M. pneumoniae* and MRMP infection status, which is significant for the rational application of antibiotics.

## Materials and methods

### Enrolled patients

We collected specimens from seven regions, including Beijing, Shenyang, Changsha, Changchun, Shanghai, Wuhan, and Gansu, from January 2013 to December 2019. Both outpatients and inpatients are included, and the inclusion criteria were as follows: (a) fever; (b) cough, sore throat, or other symptoms of respiratory tract infections; (c) the course of illness lasts 1 to 7 days; and (d) the white blood cell count is 4.0–12.0 × 10^9^/L. The exclusion criteria were as follows: (a) parent or child who refused to be tested and (b) a bacterial or viral infection with a precise etiological diagnosis. The clinical pneumonia diagnostic criteria were [1] abnormal pulmonary signs: (i) new cough or aggravation of existing respiratory disease symptoms with or without sputum, chest pain, dyspnoea, or haemoptysis; (ii) fever; and (iii) solid lung changes or wet rales with or without radiographic evidence; and [2] chest radiograph showing new patchy infiltrative shadows, lobar or segmental solid changes, hyaline of hairy glass, or interstitial changes with or without pleural effusion. Severe pneumonia was diagnosed if there were signs of severe pneumonia such as ultra-high or persistent fever, dyspnoea, lung lesions involving >2/3 of the lung tissue, and combined complications [7], such as pleural effusion and myocardial or liver damage. For all cases, the age, sex, and clinical diagnosis of the patients were collected, and the year and month of collection were recorded. Oral consent from the parents or guardians of the children in this study was approved by the Ethics Committee of Beijing Friendship Hospital (No. 2019-P2–176-02).

### Specimen processing and deoxyribonucleic acid (DNA) extraction

Respiratory samples were collected from all children, including some alveolar lavage fluid from hospitalized children, and most were pharyngeal swabs. All the samples from adult patients are pharyngeal swabs. For some children who included both pharyngeal swabs and alveolar lavage fluid, alveolar lavage fluid was used as the primary sample, and duplicate sample information was excluded and counted as one case. The collected respiratory samples were immediately placed in 2 ml of pleuropneumonia-like organism (PPLO) transfer medium (as described before [8]) and stored at −20 ~ −80 °C until transfer. The transfer was carried out by cold chain transport to the Beijing Tropical Medicine Research Institute, Beijing Friendship Hospital in Beijing, China, where it was uniformly processed by professional staff. The DNA extraction was performed on the original fluid portion after thawing, using the Universal Genomic DNA Kit (CoWin Biosciences, Jiangsu, China) following the manufacturer’s instructions, and the rest was cultured.

### Detection of *M. pneumoniae* and MRMP by polymerase chain reaction (PCR) sequencing

They were tested by PCR and sequenced for the detection of macrolide resistance genes in domain Vof 23S ribosomal ribonucleic acid (rRNA), and the primer was as before [9, 10]. If the sequence was successful, this strain was defined as PCR positive, and then, the sequencing results were compared with the reference strain amplification of *M. pneumoniae* (FH, ATCC 15531, recorded on the National Center for Biotechnology Information) using BioEdit software.

### Culture and detection of MIC

The throat swab and bronchoalveolar lavage fluid specimens were inoculated in *M. pneumoniae* liquid medium, mixed evenly, and placed in an incubator at 37 °C with 5% CO_2_ for culture, and *M. pneumoniae* growth caused a decrease in the medium’s pH that was indicated by a colour change (from red to yellow) [8]. If there was no colour change for more than one month, the culture was defined as negative. The preparation method was as described in reference [10]. Antimicrobial susceptibility testing was carried out using a broth microdilution method according to standard operating procedures from the Clinical and Laboratory Standards Institute (CLSI document M43A), and when the growth control well first showed a colour change, the lowest concentration of antibiotic in wells without colour change was defined as MIC [11]. Erythromycin, azithromycin, tetracycline, and levofloxacin were used [9]; *M. pneumoniae* reference strain FH (ATCC 15531) was used as a drug-susceptible control strain, and all tests were performed in triplicate. According to the latest version of CSLI M43-A [11], the MIC of erythromycin and azithromycin ≤0.5 μg/ml is defined as sensitive, tetracycline is ≤2 μg/ml, and levofloxacin is ≤1 μg/ml. The resistant MIC of erythromycin and azithromycin is ≥1 μg/ml. There is no definition for tetracycline and fluoroquinolone resistance.

### Data management and statistical analysis

Statistical analysis was performed using IBM Statistical Package for the Social Sciences (SPSS) software, version 25.0, for Windows (SPSS, Chicago, IL, USA). Continuous data were compared using the Mann–Whitney U-test or Student’s t-test. The differences in the categorical variables were assessed using the chi-squared or Fisher’s exact test. A *P*-value of less than 0.05 was considered statistically significant.

## Results

### Specimen summary

A total of 4,145 respiratory samples were collected in this study, including 3,916 samples from children (<18 years old) and 229 samples from adults; 3,580 were pharyngeal swab samples, and 565 were alveolar lavage fluid. A total of 19 hospitals from seven regions of China participated. The following were the hospitals from Beijing: Beijing Children’s Hospital Affiliated with Capital Medical University, Civil Aviation General Hospital, Peking University Third Hospital, China Meitan General Hospital, Beijing Chaoyang Hospital, Changping District Integrated Traditional Chinese and Western Medicine Hospital, New Century International Children’s Hospital, Peking University First Hospital, Dongfang Affiliated Hospital of Beijing University of Chinese Medicine, the First Hospital of Tsinghua University, China-Japan Friendship Hospital, Beijing Friendship Hospital, and Xiyuan Hospital of China Academy of Chinese Medical Sciences (CACMS). Here were the hospitals from other regions: Shengjing Hospital of China Medical University, Shenyang, Liaoning; the Second People’s Hospital of Hunan Province, Changsha, Hunan; the First Hospital of Jilin University, Changchun, Jilin; Shanghai Children’s Medical Center, Shanghai; and the Hubei Provincial Hospital of Traditional Chinese Medicine, Wuhan, Hubei. The respiratory samples of outpatients were all pharyngeal swabs, and some inpatients had two samples of pharyngeal swabs and alveolar lavage fluid, and the results of alveolar lavage fluid were selected and counted as one case. Individual children with incomplete information on age and sex were not included in the statistics of the corresponding results.

### Prevalence of *M. pneumoniae* and MRMP in different regions over different years

The results of PCR testing and drug resistance statistics of 789 children’s respiratory samples from different regions in 2013 showed that the highest positive rate of MRMP testing was in Beijing with 74.5%, followed by Shanghai and Liaoning with 50%, Jilin with 41.3%, Hubei with 41.2%, Gansu with 38.5%, and Hunan with 19.6% in that order. The results of the MRMP testing showed that Shanghai had the highest resistance rate of 100%, followed by Liaoning of 84.2%, Hunan of 83.3%, Beijing of 75.8%, Jilin of 73.7%, Hubei of 71.4%, and Gansu of 20% (Table 1). 23S rRNA region V sequencing results were seen for A2063G and A2064G point mutations, or both mutations, with A2063G accounting for 96% of all mutated strains. Annual analysis of 3,543 childhood respiratory infection samples collected in Beijing from January 2013 to December 2019 showed that the highest positive *M. pneumoniae* detection rate was 74.5% in the year 2013, the lowest detection rate was 32.9% in 2017, and the remaining years were 41.7% in 2014, 47.8% in 2015, 44.4% in 2016, 53.1% in 2018, and 58.1% in 2019, respectively. MRMP results showed the lowest total drug resistance detection rate was 70.2% in 2014, the highest was 97.4% in 2019, and the remaining years were 75.8% in 2013, 71.2% in 2015, 74.3% in 2016, 84.8% in 2017, and 94% in 2018 (Table 2). All adult samples were collected from 2016, 103 males and 126 females, and the *M. pneumoniae* test results for all samples showed 41 positive samples, with a positive test rate of 17.9%, including 24 cases containing the A2063G point mutation and 17 cases without drug-resistant mutations, with a resistance rate of 10.48%, and the positive test rate and resistance rate were lower than those of children.

**Table 1.** Prevalence of *Mycoplasma pneumoniae* and MRMP in different regions, in the year 2013

	PCR-positive rate				Macrolide-resistant rate^a^			
	Positive	Negative	Total	Positive rate	MRMP	MSMP	Total	Resistant rate
Changchun, Jilin	19	27	46	41.3%	14	5	19	73.7%
Xiahe, Gansu	5	8	13	38.5%	1	4	5	20.0%
Shanghai	22	22	44	50.0%	22	0	22	100.0%
Wuhan, Hubei	7	10	17	41.2%	5	2	7	71.4%
Changsha, Hunan	10	33	51	19.6%	15	3	18	83.3%
Shenyang, Liaoning	101	101	202	50.0%	85	16	101	84.2%
Beijing	310	106	416	74.5%	235	75	310	75.8%
Total	474	307	789	60.1%	377	105	482	78.2%

Abbreviations: MRMP, macrolide-resistant *M. pneumoniae*; MSMP, macrolide-sensitive *M. pneumoniae.*

a The data shown in this table are the PCR sequencing results of DNA extracted directly from the original respiratory samples.

**Table 2.** Prevalence characteristics of *Mycoplasma pneumoniae* and MRMP between inpatients and outpatients over the years

	PCR-positive rate						Macrolide-resistant rate				
Year		Positive(total)	Positive rate	Positive in all	χ^2^	*P* value	MR (total)	Resistant rate	Resistant in all	χ^2^	*P* value
2013	Inpatient	160(200)	80.00%	74.50%	6.094	0.014	138(160)	86.30%	75.80%	19.664	<0.01
	Outpatient	150(216)	69.40%				97(150)	64.70%			
2014	Inpatient	163(277)	58.80%	41.70%	51.94	<0.01	130(163)	79.80%	70.20%	14.104	<0.01
	Outpatient	163(505)	32.30%				99(163)	60.70%			
2015	Inpatient	47(79)	59.50%	47.80%	10.081	0.01	31(47)	66.00%	71.20%	2.199	0.138
	Outpatient	19(59)	32.20%				16(19)	84.20%			
2016	Inpatient	153(274)	55.80%	44.40%	12.117	<0.01	129(153)	84.30%	74.30%	6.895	<0.01
	Outpatient	139(383)	36.30%				88(139)	63.30%			
2017	Inpatient	127(272)	46.70%	32.90%	59.595	<0.01	118(127)	92.90%	84.80%	14.188	<0.01
	Outpatient	51(269)	19.00%				33(51)	64.70%			
2018	Inpatient	222(379)	58.60%	53.10%	23.218	<0.01	212(222)	95.50%	94.00%	6.983	<0.01
	Outpatient	30(96)	31.30%				25(30)	83.30%			
2019	Inpatient	283(385)	73.50%	58.10%	135.324	<0.01	278(283)	98.20%	97.40%	8.561	<0.01
	Outpatient	27(149)	18.10%				24(27)	88.90%			
Total	Inpatient	1,155(1866)	61.90%	48.90%	360.21	<0.01	1,036(1155)	89.70%	81.80%	210.46	<0.01
	Outpatient	579(1677)	34.50%				382(579)	66.00%			

*Note:* The data shown in this table are the PCR sequencing results of DNA extracted directly from the original respiratory samples. The total samples were 3,543, including 1866 from inpatients and 1,677 from outpatients.

Abbreviations: MRMP, macrolide-resistant *M. pneumoniae*; MSMP, macrolide-sensitive *M. pneumoniae.*

### Prevalence of *M. pneumoniae* and MRMP in different age groups

The enrolled children were divided into three groups: toddlers (0–3 years old), pre-school children (4–6 years old), and school-age children (over 7 years old) for comparative analysis of *M. pneumoniae* and MRMP in different age groups. The results showed that the positive rate of *M. pneumoniae* in school-age children was 66.8%, which was higher than 31.1% in toddlers and 47.6% in pre-school children, and the difference was statistically significant (*P* < 0.01). The macrolide-resistant rate in the school-age group was 86.9%, higher than 73.5% in the toddlers and 79.5% in the pre-school group, and the difference was statistically significant (*P* < 0.01).

### Characteristics of *M. pneumoniae* and MRMP by genders

The positive rate of *M. pneumoniae* in children between genders showed that 48.2% of males tested positive and 42.1% of females with no significant difference (*P* = 0.236); the macrolide-resistant rate of *M. pneumoniae* showed 79.9% of males and 98.3% of females, also with no significant difference (*P* = 0.067).

### Prevalence characteristics of *M. pneumoniae* and MRMP between inpatients and outpatients

**Figure 1.**
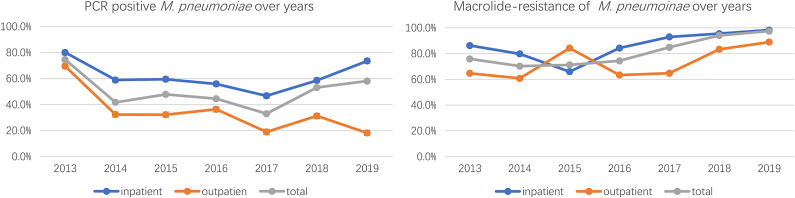
PCR positive and macrolide-resistance of M. pneumoniae from 2013-2019.

The analysis of *M. pneumoniae* and MRMP between outpatients and inpatients was conducted in cases from Beijing and Shenyang, a total of 3,543 samples were included, and the overall *M. pneumoniae* positive rate was 48.9%, of which a total of 1,677 outpatient medical records with a PCR-positive rate of 34.5% and a total of 1866 inpatient samples with a PCR-positive rate of 61.9% were included, and the PCR-positive rate of hospitalized children was significantly higher than that of the outpatient ones, and the difference was significant (*P* < 0.01). Among *M. pneumoniae*-positive samples, there were 1734 cases of MRMP with a total resistance rate of 81.8%, among which 382 cases were from outpatients with a macrolide-resistant rate of 66% and 1,036 cases were from inpatients with a macrolide-resistant rate of 89.7%. MRMP was more prevalent in patients who were admitted to the hospital, and the difference was significant (*P* < 0.01) (Table 2 and Figure 1).

### Clinical characteristics of *M. pneumoniae* and MRMP

Among all children hospitalized for CAP, the PCR-positive rate of *M. pneumoniae* was higher in children with severe pneumonia than in children with common pneumonia, 76.8% vs. 55.2%, respectively, with a significant difference (*P* < 0.01). Besides, the macrolide-resistant rate was significantly higher in severe pneumonia than in common pneumonia, 93.9% vs. 88.1%, respectively, with significant differences (*P* < 0.01) (Table 3).

**Table 3.** Clinical characteristics of *Mycoplasma pneumoniae* and MRMP

PCR-positive rate							Macrolide-resistant rate				
Common pneumonia		Severe pneumonia		*P* value	Common pneumonia			Severe pneumonia			*P* value
Positive number (total number)	Positive rate	Positive number (total number)	Positive rate		MRMP	MSMP	Resistant rate	MRMP	MSMP	Resistant rate	
573(746)	76.8%	1,205(2183)	55.2%	<0.01	538	35	93.9%	1,062	143	88.1%	<0.01

### MICs of antibiotics

All the collected respiratory tract samples were cultured, a total of 481 *M. pneumoniae* clinical strains were isolated, and 45 clinical isolates were selected for MIC detection of four antibiotics as follows: erythromycin, azithromycin, tetracycline, and levofloxacin. The MIC values of all MRMP to erythromycin and azithromycin were ≥ 64 μg/ml and ≥ 32 μg/ml, respectively. The MICs of tetracycline and levofloxacin were ≤ 0.5 μg/ml and ≤ 1 μg/ml, respectively (Supplemental file).

## Discussion

*M. pneumoniae* is an important pathogen of respiratory infections in children and adults, accounting for about 10–40% of CAP in children, with an epidemic every 4–7 years [4, 12]. *M. pneumoniae* is mostly droplet-borne and is more likely to spread in relatively closed population centres such as schools and military units [13]. People of all ages are susceptible, and clinical manifestations vary, can be self-limiting, or can develop into severe cases, causing multiple complications within and outside the respiratory system. *M. pneumoniae* lacks a cell wall, which makes it naturally resistant to antibiotics that act on the cell wall. Considering children are in a special period of growth and development, macrolide antibiotics are the first-line antibiotics for clinical treatment of *M. pneumoniae* infection in childhood, and tetracycline and quinolone antibiotics are restricted in use in children [14]. With the use of antibiotics, *M. pneumoniae* faces tremendous pressure for drug selection. Since the first report of MRMP clinical isolates in Japan in 2000, countries have reported successively. However, the resistance rate varies widely among different countries and regions, and the resistance rate is generally high in China. Point mutations in domain V of the 23S rRNA of *M. pneumoniae* cause drug resistance by affecting the action site of macrolide antibiotics, which is the main resistance mechanism, and no other mechanisms related to drug resistance have been found [15].

In our study, analysis of the prevalence and macrolide resistance of *M. pneumoniae* in some domestic regions in 2013 showed that the highest positive PCR testing rate was in Beijing (74.5%), followed by Shanghai (50%), Shenyang (50%), Changchun (41.3%), Wuhan (41.2%), Xiahe, (38.5%), and Changsha (19.6%), while the macrolide resistance showed that Shanghai had the highest resistance rate with 100%, while Gansu has the lowest resistance rate of 20%. Macrolide resistance varied among different regions, Xue et al. reported that the resistant rate was 86.7% in Beijing, 81.8% in Shanghai, 74.3% in Kunming, 66.7% in Harbin, 80% in Urumqi, and 20% in Nanjing [16], but the number of samples from Nanjing is only ten. Xu et al. reported that macrolide-resistant rate of *M. pneumoniae* is 92.39% in children, from 2014 to 2016, in Nanjing [17]. Jiang et al. reported the *M. pneumoniae* epidemic in Qingdao, with a positive *M. pneumoniae* test rate of 59% for respiratory tract infections in children, of which the macrolide-resistant rate was as high as 100% [18], and the PCR-positive rate of *M. pneumoniae* in children reported in Weihai during 2019 was 88.1%, and the macrolide-resistant rate was 98.78% [19]. These reports are basically consistent with those in our paper. This study is the first to report on the Hunan, Hubei, and Gansu regions. The prevalence and drug resistance of *M. pneumoniae* vary greatly in different regions and years. *M. pneumoniae* is a respiratory pathogen that is transmitted by droplet transmission, correlates with urban population density and population mobility, and is influenced by temperature, humidity, and other climatic factors. Xiahe, a small county in Gansu, China, has a sparsely populated area and a dry climate, which may have contributed to the low rate of *M. pneumoniae* infection and drug resistance. Although the *M. pneumoniae-*positive rate and MRMP rate in Kunming are at a high level, *M. pneumoniae* is not a common pathogen in the southern areas and has rarely been reported in Guangdong, Guangxi, and Fujian provinces. As a first-tier city in China, there is no report of the *M. pneumoniae* outbreak in children in Guangzhou, but in Beijing, *M. pneumoniae* is one of the major pathogens of respiratory infections in children, especially in the fall and winter. There is a regional disparity in *M. pneumoniae* infection and drug resistance rates. With limited medical resources, it is of great significance to increase the surveillance of *M. pneumoniae* and MRMP in specific areas to provide early warning for clinics.

In this paper, the age of children ranged from 1 month to 18 years, but the number of children younger than 1 year old and older than 14 years old was both small, with less than 10 cases. All cases were divided into three groups: toddler group, pre-school group, and school-age group according to different age stages, and the prevalence and infection of *M. pneumoniae* were analysed and studied, and the results showed that school-age children had a higher PCR-positive rate of *M. pneumoniae* and macrolide resistance than the other two groups of children in both outpatient and inpatient children, and the difference was significant. There was no significant correlation between *M. pneumoniae* infection and drug-resistant rates in children of different genders, independent of disease severity susceptibility, which is consistent with previous reports [20, 21].

Comparative analysis of medical records collected from outpatients and inpatients showed that the rate of positive PCR tests was significantly higher in ward children than in outpatient children, 61.9% vs. 34.5%, and the rate of drug resistance was also higher than in outpatient clinics, 89.7% vs. 66%, both significantly different. Compared with common pneumonia, the severe ones had a higher positive rate of testing and drug resistance. The results of our *M. pneumoniae* resistance testing of inpatients with severe and general pneumonia in 2019 showed that the fever course, duration of antibiotic treatment, and length of hospital stay may be longer for infections with drug-resistant strains. The pathogenic mechanism of *M. pneumoniae* is complex and includes multiple aspects such as direct damage, toxin damage, and immune damage [22]. Although there are no studies showing that drug-resistant strains are directly correlated with clinical manifestation, it is shown that the rapid and massive multiplication of *M. pneumoniae* in the alveoli could cause direct tissue damage and blood exposure, which stimulates the body’s immune response, contributing to neutrophil and cytokine expression, which may in turn exacerbate the disease [23, 24]. Children infected with resistant strains transferring antibiotics to tetracyclines or fluoroquinolones could significantly shorten the length of hospitalization and fever duration [25, 26], and it is important to use glucocorticoids promptly to modulate immunity [27]. However, analysis of the clinical characteristics of MRMP infections in the United States showed no statistical difference [28]. Studies with multicentre and adequate clinical samples are needed.

**Figure 2.**
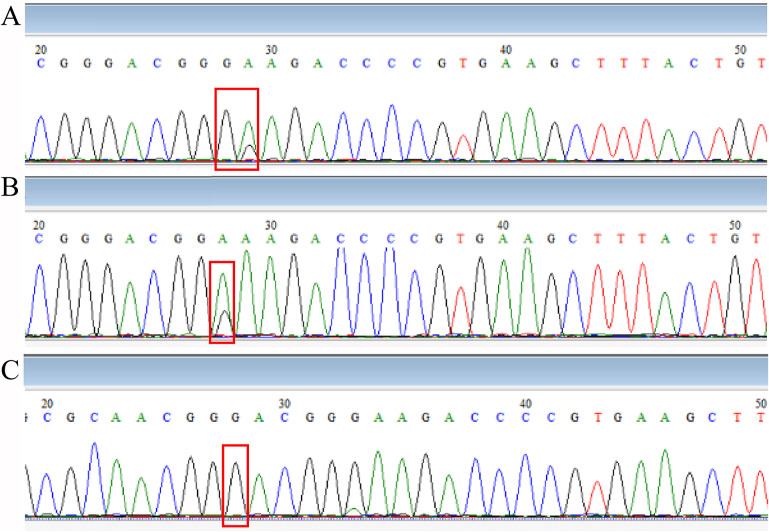
Comparison of sequencing results. Note: (A: Combination of M. pneumoniae with 2063 A→G point mutation, with or without 2064 point mutation; B: Combination of M. pneumoniae with or without 2063 A→G point mutation; C: M. pneumoniae with 2063 A→G point mutation; position 28 is 2063 point and position 29 is 2064 point from domain V of 23S rRNA.).

The sequencing results of drug-resistant strains showed that A2063G was the main drug-resistant mutation, accounting for 93.7%, and A2064G accounted for 4.5%, 1.8% combined with the A2063G + A2064G mutation. The MIC results of commonly used antibiotics on clinical isolates showed that the resistant strains generally had a high level of resistance to erythromycin and azithromycin of macrolides (MIC≥256 μg/ml) and no resistance to tetracycline and levofloxacin (MICs were less than 1 μg/ml). The mutant-free strain was <1 μg/ml for erythromycin and azithromycin MIC. This is the same as reported in previous studies. The development of resistance in *M. pneumoniae* may be related to the pressure of antibiotics. There are different types of macrolides, containing different metacyclic rings, and the loci may be slightly different. It has been reported in Japan that the MIC resistance value of the A2064G point mutant strain to erythromycin is lower than that caused by A2063G [29]. But strains containing mutations at position 2063 and/or 2064 have not been successfully isolated. As shown in Figure 2, sequencing of DNA extracted directly from pharyngeal swabs showed that in vivo, susceptible, and resistant strains may coexist, and it may be associated with exposure to different antibiotics. However, since we did not isolate a strain containing both mutations after purification, whether this is a single strain with both mutations or a mixed infection is unknown and requires further study.

Limitations of this article: The article only collected samples and data from some regions of the country in 2013, and the samples were mainly from Beijing and Shenyang, with little data from the central and western regions, which has an impact on our understanding of the current situation of infection in the central and western regions, and most of the data in this article came from the provincial capitals of the regions, which are insufficient for understanding the infection and prevalence at the grassroot level. We should go further to promote the surveillance situation of *M. pneumoniae* and MRMP in multicentre and multidisciplinary centres nationwide and promote the clinical routine testing of MRMP, especially in the eastern regions where MRMP is prevalent, which is significant for clinical early warning work.

## Supporting information

Jiang et al. supplementary material Jiang et al. supplementary material

## Data Availability

The datasets are not publicly available due to their containing personal data but will be made available by the corresponding author upon reasonable request .

## Supplementary material

For supplementary material accompanying this paper visit http://doi.org/10.1017/S0950268824000323. click here to view supplementary material
