# Supplementary material for: Macrolide resistance of Mycoplasma pneumoniae in several regions of China from 2013 to 2019
Source: Epidemiol Infect. 2024 Apr 18;152:e75. doi: 10.1017/S0950268824000323 (PMC11094376; doi:10.1017/S0950268824000323)
Supplement: Jiang et al. supplementary material [file S0950268824000323sup001.docx]

Table 4 MICs of *M. pneumoniae* isolates

| Year | **Name** | Resistant mutation | MIC(ug/ml) | | | |
| --- | --- | --- | --- | --- | --- | --- |
|  |  |  | Erythromycin | Azithromycin | Levofloxacin | Tetracycline |
| 2015 | CYM135 | A2063G | 512 | 256 | 1 | 0.125 |
|  | CYM156 | A2063G | 1024 | 512 | 1 | 0.125 |
| 2016 | CYM165 | A2063G | 512 | 256 | 0.5 | 0.06 |
|  | CYM170 | A2063G | 256 | 64 | 0.2 | 0.40 |
|  | CYM173 | A2063G | 256 | 64 | 0.2 | 0.40 |
|  | CYM174 | A2063G | 128 | 32 | 0.2 | 0.40 |
|  | CYM178 | A2063G | 512 | 256 | 1 | 0.125 |
|  | CYM207 | A2063G | 256 | 32 | 0.125 | 0.25 |
|  | CYM208 | A2063G | 512 | 512 | 1 | 1.00 |
|  | CYM215 | A2063G | 1024 | 512 | 1 | 0.50 |
|  | CYM217 | A2063G | 512 | 512 | 1 | 0.25 |
|  | CYM219 | A2063G | 512 | 128 | 0.5 | 0.125 |
|  | CYM227 | A2063G | 512 | 256 | 1 | 0.13 |
|  | CYM228 | A2063G | 512 | 128 | 0.5 | 0.25 |
| 2017 | CYM234 | A2063G | 512 | 256 | 1 | 0.13 |
|  | CYM235 | A2063G | 512 | 128 | 0.5 | 0.25 |
|  | CYM266 | A2063G | 1024 | 256 | 1 | 0.125 |
|  | CYM267 | A2063G | 64 | 256 | 1 | 0.125 |
|  | CYM273 | A2063G | 128 | 1024 | 1 | 0.125 |
|  | CYM277 | A2063G | 1024 | 256 | 1 | 0.125 |
|  | CYM282 | A2063G | 512 | 256 | 1 | 0.13 |
|  | CYM283 | A2063G | 512 | 128 | 1 | 0.25 |
|  | CYM317 | A2063G | 512 | 256 | 1 | 0.25 |
| 2018 | CYM329 | A2063G | 1024 | 256 | 0.5 | 0.13 |
|  | CYM330 | A2063G | 512 | 256 | 0.5 | 0.06 |
|  | CYM331 | A2063G | 512 | 256 | 0.5 | 0.13 |
|  | CYM332 | A2063G | 512 | 512 | 0.5 | 0.06 |
|  | CYM334 | A2063G | 512 | 256 | 0.5 | 0.06 |
|  | CYM339 | A2063G | 512 | 256 | 0.5 | 0.06 |
|  | CYM340 | A2063G | 1024 | 256 | 1 | 0.25 |
|  | CYM347 | A2063G | 512 | 256 | 1 | 0.25 |
|  | CYM366 | A2063G | 512 | 128 | 0.5 | 0.06 |
|  | CYM378 | A2063G | 512 | 256 | 0.5 | 0.06 |
|  | CYM380 | A2063G | 1024 | 256 | 1 | 0.25 |
| 2019 | CYM384 | A2063G | 512 | 256 | 1 | 0.25 |
|  | CYM387 | A2063G | 512 | 512 | 1 | 0.25 |
|  | CYM388 | A2063G | 1024 | 128 | 1 | 0.25 |
|  | CYM389 | A2063G | 1024 | 256 | 0.5 | 0.06 |
|  | CYM391 | A2063G | 1024 | 128 | 1 | 0.06 |
|  | CYM394 | A2063G | 1024 | 256 | 0.125 | 0.50 |
|  | CYM397 | A2063G | 1024 | 128 | 1 | 0.06 |
|  | CYM400 | A2063G | 1024 | 512 | 1 | 0.25 |
|  | CYM401 | A2063G | 1024 | 128 | 1 | 0.25 |
|  | CYM403 | A2063G | 512 | 128 | 1 | 0.25 |
|  | CYM406 | A2063G | 1024 | 256 | 1 | 0.25 |

*note: MIC=Minimum inhibition concentration; Erythromycin and Azithromycin belong to macrolides; Levofloxacin belongs to fluoroquinolones;
